# Additive Effect of Anemia and Renal Impairment on Long-Term Outcome after Percutaneous Coronary Intervention

**DOI:** 10.1371/journal.pone.0114846

**Published:** 2014-12-09

**Authors:** Thomas Pilgrim, Martina Rothenbühler, Bindu Kalesan, Cédric Pulver, Giulio G. Stefanini, Thomas Zanchin, Lorenz Räber, Stefan Stortecky, Simon Jung, Heinrich Mattle, Aris Moschovitis, Peter Wenaweser, Bernhard Meier, Thomas Gsponer, Stephan Windecker, Peter Jüni

**Affiliations:** 1 Department of Cardiology, Swiss Cardiovascular Center, Bern University Hospital, Bern, Switzerland; 2 Institute of Social and Preventive Medicine and Clinical Trials Unit, University of Bern, Bern, Switzerland; 3 Department of Neurology, Bern University Hospital, Bern, Switzerland; University of Munich, Germany

## Abstract

**Introduction:**

Anemia and renal impairment are important co-morbidities among patients with coronary artery disease undergoing Percutaneous Coronary Intervention (PCI). Disease progression to eventual death can be understood as the combined effect of baseline characteristics and intermediate outcomes.

**Methods:**

Using data from a prospective cohort study, we investigated clinical pathways reflecting the transitions from PCI through intermediate ischemic or hemorrhagic events to all-cause mortality in a multi-state analysis as a function of anemia (hemoglobin concentration <120 g/l and <130 g/l, for women and men, respectively) and renal impairment (creatinine clearance <60 ml/min) at baseline.

**Results:**

Among 6029 patients undergoing PCI, anemia and renal impairment were observed isolated or in combination in 990 (16.4%), 384 (6.4%), and 309 (5.1%) patients, respectively. The most frequent transition was from PCI to death (6.7%, 95% CI 6.1–7.3), followed by ischemic events (4.8%, 95 CI 4.3–5.4) and bleeding (3.4%, 95% CI 3.0–3.9). Among patients with both anemia and renal impairment, the risk of death was increased 4-fold as compared to the reference group (HR 3.9, 95% CI 2.9–5.4) and roughly doubled as compared to patients with either anemia (HR 1.7, 95% CI 1.3–2.2) or renal impairment (HR 2.1, 95% CI 1.5–2.9) alone. Hazard ratios indicated an increased risk of bleeding in all three groups compared to patients with neither anemia nor renal impairment.

**Conclusions:**

Applying a multi-state model we found evidence for a gradient of risk for the composite of bleeding, ischemic events, or death as a function of hemoglobin value and estimated glomerular filtration rate at baseline.

## Introduction

Patients with coronary artery disease undergoing revascularization are often found to have anemia and renal impairment. Both conditions are independently associated with increased risk of bleeding, ischemic events, and mortality [1]–[9]. Anemia and renal impairment may be directly causally related, or they may be a marker of underlying morbidities. Both conditions are associated with established cardiovascular risk factors, such as age, gender and diabetes, and may coexist in the absence of a direct causal relationship, which is observed in 10 to 25% of patients undergoing percutaneous coronary intervention (PCI) for stable coronary artery disease or acute coronary syndromes [1]–[3], [7]. Routine assessment of haemoglobin and glomerular filtration rate in patients undergoing PCI may not only have immediate implications for the peri-procedural management, but may also provide a useful tool to approximate progression of disease after PCI. The combined effect of anemia and renal impairment on long-term clinical outcome has not been investigated in large cohorts. Our objective was to estimate long-term mortality associated with anemia and renal impairment, separately and in combination, while also accounting for the most relevant intermediate outcomes, such as bleeding or ischemic events. We used data from a prospective registry of patients undergoing PCI with the unrestricted use of drug eluting stents (DES) to provide novel insights by quantifying transition-specific risk estimates.

## Methods

### Patient Population and Inclusion Criteria

Between April 2002 and March 2009, a total of 6300 patients underwent 6529 interventions with the unrestricted use of early and newer generation DES at Bern University Hospital, Switzerland, and were prospectively entered into the Bern DES registry. A total of 229 patients received both early and newer generation DES at two different points in time. We considered the first intervention as eligible for the reporting of the baseline characteristics and for this analysis in general. Patients who had missing information regarding the implanted stent (n = 5), missing hemoglobin values at baseline (n = 193), or had not undergone follow-up after the index procedure (n = 74) were excluded, resulting in 6029 subjects for the purpose of the present analysis.

### Data Collection

In the DES registry, demographic information and clinical characteristics such as type of stent implanted and related PCI information was collected systematically. Laboratory values were retrieved from the local central hematology and chemistry laboratory, including hemoglobin and hematocrit values on admission, as well as creatinine at baseline. In-hospital outcome data were captured from the electronic hospital records. Subsequently, data on vital status were recorded for all patients from hospital records and municipal civil registries, and a postal questionnaire was sent to all living patients with questions on re-hospitalization and major adverse cardiovascular events. Patients, general practitioners and referring cardiologists were contacted by telephone in case of non-response or if additional information was required. In case of suspected cardiovascular events, medical records, discharge letters, and coronary angiography documentation were systematically collected and reviewed.

### Ethics Statement

The registry was approved by the institutional ethics committee at Bern University Hospital, Switzerland (www.kek-bern.ch), and complied with the Declaration of Helsinki. Written informed consent was given by the patients for their information to be stored in the hospital database and used for research.

### Procedures

PCI was performed according to practice guidelines that applied at the time of stent implantation. During intervention, unfractionated heparin in a dose of 5000 IU or 70–100 IU/kg was administered targeting an activated clotting time of >250 seconds. Before or at the time of the procedure, dual antiplatelet therapy was initiated with acetylsalicylic acid ≥100 mg and clopidogrel 300–600 mg; while clopidogrel was recommended to be continued for the duration of at least 12 months, acetylsalicylic acid was prescribed lifelong. Peri-procedural administration of glycoprotein IIb/IIIa inhibitors was left to the discretion of the operator. Before and after the procedure a 12-lead electrocardiogram was obtained and cardiac enzymes were assessed within 24 hours of the procedure. In addition to routine assessment of creatinine kinase (CK), CK-MB and troponin T, repeat measurements of these markers were performed every 6–8 hours in patients with suspected ischemia until the peak level was identified.

### Definitions

Categorization of anemia was performed in accordance with the definition provided by the World Health Organization (WHO), defining anemia as a hemoglobin concentration <120 g/l in women and <130 g/l in men [10]. Patients were classified to have renal impairment if there was moderate renal insufficiency as defined by an eGFR according to the Cockroft-Gault formula of <60 ml/min [11]. Creatinine levels and weight at baseline were used to estimate glomerular filtration rate (eGFR). Bleeding events were classified according to the consensus report from the Bleeding Academic Research Consortium (BARC) definition [12]. Myocardial infarction (MI) included Q-wave and non-Q-wave MI and was diagnosed in the setting of symptoms or signs of ischemia in the presence of new pathological Q-waves in ≥2 contiguous leads, or an elevation in CK to ≥2x the upper reference limit (URL) and a rise in CK-MB, or troponin to ≥3x URL in the presence of ischemic symptoms or ECG changes. Cerebrovascular events were reviewed and adjudicated by two board certified neurologists (SJ, HM). Ischemic stroke was diagnosed in case of a focal neurological deficit (motor or sensory deficit, dysarthria, aphasia, visual loss) with duration of ≥24 hours and/or imaging documentation of ischemia and exclusion of intracranial bleeding. Ischemic events (IE) were defined as the composite of myocardial infarction or ischemic stroke. Death comprises both cardiovascular (CV) and non-cardiovascular (non-CV) death and was considered as the primary outcome throughout the present analysis. All potential events were centrally adjudicated based on relevant medical records. Only the first event of each type of clinical outcome was used for this analysis. The duration of observation was defined as time from PCI to death or end of follow-up up to four years, without censoring at the time of occurrence of an intermediate outcome.

### Statistical analysis

**Figure 1:**
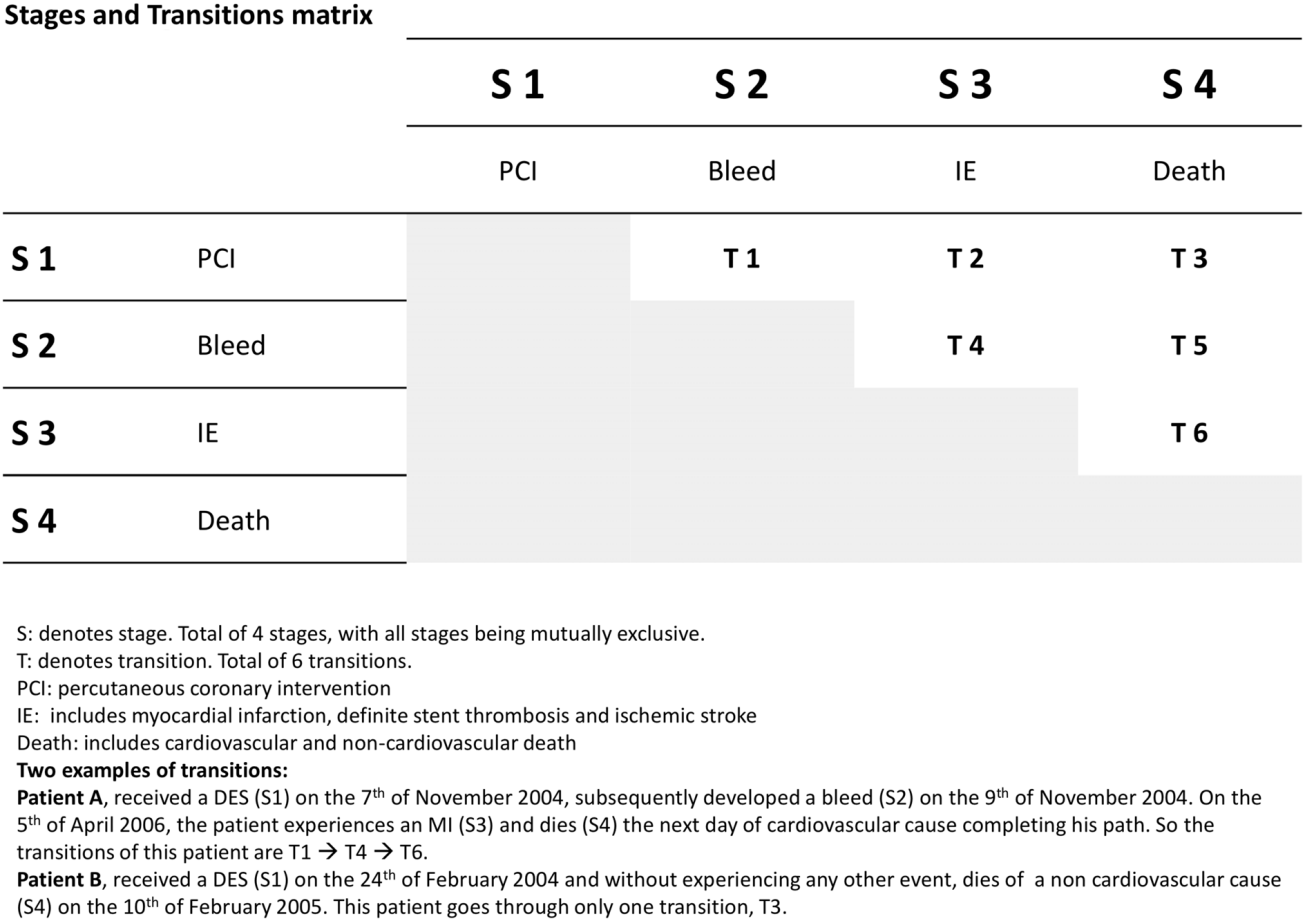
illustrates the four stages of disease included into the multi-state analysis and the six stage-to-stage transitions a patient may experience from the time of PCI to death or end of follow-up.

Disease progression to eventual death can be understood as the combined effect of baseline characteristics and intermediate outcomes. Considering both the intermediate outcomes and the final endpoint, we identified four stages (PCI, bleeding, ischemic events and death), resulting in six stage-to-stage transitions a patient could undergo from PCI to death or end of follow up. The four stages and the corresponding transitions are illustrated in Fig. 1. Transitions from PCI to bleeding, ischemic events and death were referred to as primary transitions (T1 to T3). Further transitions from bleeding to ischemic events and death were defined as secondary transitions (T4 to T6). We ignored possible recurrent transitions, e.g. from ischemic events to bleeding or re-intervention due to the low number of observations (n = 3) and the missing corresponding baseline characteristics, respectively. We performed a continuity correction in case two events occurred on the same day.

All analyses were performed in order to compare the four target subgroups: patients with anemia only, patients with renal impairment only, patients with both conditions, and patients with neither condition at baseline (reference group). We assessed the additivity of the two conditions by comparing different Cox proportional hazard models on the primary outcome using the likelihood ratio test. The first two models include only one variable, informing about the presence of either anemia or renal impairment. The third model includes both conditions while the fourth model considers additionally the interaction between the two conditions. The non-significant interaction term was interpreted as lack of evidence for a synergetic effect of the two conditions [13]–[16]. Furthermore, the additive effect of anemia and renal impairment was also estimated using a weighted random effects regression model across ordered groups. The ordering of the groups is based on the χ^2^-statistic of different Cox proportional hazard regressions of the primary outcome, death, using all possible orders of the groups.

Baseline and procedural characteristics were compared between subgroups using logistic or multinomial regression for categorical and linear regression analysis for continuous variables. We compared the number of transitions among the four subgroups using multinomial logistic regression. We estimated the hazards for each of these transitions and calculated transition-specific hazard ratios for each subgroup using Cox proportional hazard models within a multi-state analysis (MSA) [17]. MSA allows us to describe the transition from PCI through intermediate clinical outcomes (bleeding and ischemic events such as myocardial infarction, ischemic stroke and definite stent thrombosis) to eventual death. We use MSA to estimate all-cause mortality because the intermediate events might change the risk of the endpoint to occur and can be treated as competing intermediate risks. The intermediate events provide more information on the disease progression and considering them allows for more precision in predicting the disease progression [18]. The model allowed for different baseline hazards for each transition and adjusted for type of stent (early versus newer generation DES), age, gender, diabetes, hypertension, dyslipidemia, left ventricular ejection function (LVEF) and acute coronary syndrome (ACS). Results are presented as transition-specific hazard ratios (HR) with 95% confidence intervals (CI). Based on transition-specific hazards, we predicted the transition probability, defined as the probability of a particular event at a given time point for the four target subgroups. We estimate the trend using linear weighted random effects. Analyses were performed using R version 3.1 (The R Development Core Team, Vienna, Austria) using the mstate package [17] and STATA version 13.1.

## Results

A total of 6029 subjects undergoing PCI with the unrestricted use of early and newer generation DES were considered for the present analysis. Both anemia and renal impairment were present in 309 (5.1%), anemia only in 990 (16.4%), renal impairment only in 384 (6.4%), and neither anemia nor renal impairment (reference group) in 4346 patients (72.1%). The median follow-up of the overall cohort amounted to 3.2 years (interquartile range [IQR] 2.3–4.1 years). The median follow-up was 3.2 years (IQR 2.4–4.1) in the reference group, 3.0 years (IQR 2.1–4.0) among patients with anemia only, 3.3 years (IQR 2.3–4.1) among patients with renal impairment only, and 2.7 years (IQR 1.7–3.8) among patients with both anemia and renal impairment. The association between the subgroups and the median duration of follow-up was statistically significant (p<0.001).

### Baseline and procedural characteristics

Baseline characteristics are summarized in Table 1, with statistically significant differences between all characteristics across subgroups. Patients in the two subgroups with renal impairment (with and without anemia) were older, more often female and had a higher prevalence of hypertension. Table 2 presents procedural characteristics.

**Table 1:** Baseline characteristics.

Baseline characteristics	Reference	Anemia only	Renal impairment only	Both anemia and renal impairment	p-value for differences across groups
Total (n)	4346	990	384	309	
Age (yr [SD])	61.8 (11.3)	66.3 (11.1)	71.5 (9.3)	72.4 (10.1)	<0.001
Men (n [%])	3445 (79.3)	716 (72.3)	224 (58.3)	163 (52.8)	<0.001
BMI (mean [SD])	27.4 (4.2)	26.4 (4.4)	27.8 (4.8)	27.6 (4.8)	<0.001
Hypertension (n [%])	2407 (55.5)	575 (58.6)	273 (71.5)	221 (71.8)	<0.001
Dyslipidemia (n [%])	2401 (55.4)	487 (49.6)	203 (53.1)	137 (44.5)	<0.001
Diabetes mellitus (n [%])	644 (14.9)	227 (23.1)	92 (24.1)	99 (32.1)	<0.001
Smoking at baseline (n [%])	2385 (55.0)	463 (47.1)	147 (38.5)	103 (33.4)	<0.001
Left ventricular ejection fraction, <30% (n [%])	77 (1.8)	28 (3.1)	18 (5.2)	17 (6.5)	<0.001
Acute coronary syndrome (n [%])	2281 (52.5)	640 (64.6)	163 (42.6)	152 (49.2)	<0.001
ST-elevation MI (n [%])	1202 (52.8)	365 (57.0)	91 (56.2)	63 (41.7)	0.006
Second generation stent (n [%])	1450 (33.4)	406 (41.0)	110 (28.6)	131 (42.4)	<0.001
Type of stent (n [%])					<0.001
PES	961 (22.1)	186 (18.8)	89 (23.2)	58 (18.8)	
SES	1935 (44.5)	398 (40.2)	185 (48.2)	120 (38.8)	
ZES	498 (11.5)	107 (10.8)	41 (10.7)	44 (14.2)	
EES	952 (21.9)	299 (30.2)	69 (18.0)	87 (28.2)	
Hgb on admission (mean [SD])	144.5 (11.2)	115.6 (12.0)	140.5 (11.6)	109.8 (11.8)	<0.001
Htc on admission (mean [SD])	0.4 (1.0)	0.3 (0.0)	0.4 (0.0)	0.3 (0.0)	0.001
Creatinine (mean [SD])	76.4 (14.6)	75.7 (16.1)	133.5 (78.0)	175.9 (136.0)	<0.001

Reference category: No anemia and no renal impairment.

**Table 2:** Procedural characteristics.

Proceduralcharacteristics	Reference	Anemiaonly	Renalimpairmentonly	Both anemiaand renalimpairment	p-value for differencesacross groups
Total (n)	4346	990	384	309	
Multivesseltreatment (n [%])	829 (19.1)	193 (19.5)	64 (16.7)	76 (24.8)	0.054
Number of vesselstreated per patient (n [SD])	1.2 (0.4)	1.2 (0.4)	1.2 (0.4)	1.3 (0.5)	0.72
Number of lesionstreated per patient (n [SD])	1.6 (0.8)	1.6 (0.9)	1.5 (0.8)	1.7 (0.9)	0.121
Target vessel - numberof patients (n [%])					
Left main	89 (2.1)	43 (11.2)	17 (4.4)	23 (7.5)	<0.001
Left anteriordescending	2181 (50.3)	446 (45.1)	196 (51.0)	146 (47.6)	0.025
Left circumflex	1148 (26.5)	254 (25.7)	86 (22.4)	87 (28.3)	0.276
Right coronary artery	1494 (34.4)	401 (40.6)	127 (33.1)	101 (32.9)	0.002
Arterial bypass graft	9 (0.2)	3 (0.3)	0 (0.0)	2 (0.7)	0.33
Saphenous vein graft	115 (2.7)	25 (2.5)	12 (3.1)	15 (4.9)	0.136
Number of stentsimplanted (n [SD])	1.8 (1.0)	1.8 (1.0)	1.7 (1.0)	1.9 (1.1)	0.176
Average stentdiameter (n [SD])	2.9 (0.5)	2.9 (0.4)	2.8 (0.4)	2.8 (0.4)	0.011
Total stent lengthper patient (n [SD])	29.9 (18.0)	31.1 (19.0)	28.7 (17.1)	32.6 (20.6)	0.008
Glycoprotein IIb/IIIaantagonist (n [%])	993 (22.8)	276 (27.9)	49 (12.8)	43 (13.9)	<0.001
Medication atdischarge (n [%])					
Aspirin	4217 (97.1)	954 (96.4)	354 (92.9)	283 (92.2)	<0.001
Clopidogrel	4254 (98.2)	965 (97.8)	360 (94.5)	290 (95.1)	<0.001
Oral anticoagulation	71 (1.6)	21 (2.1)	17 (4.5)	13 (4.3)	<0.001
Betablocker	2681 (61.9)	623 (63.1)	228 (59.8)	185 (60.7)	0.681
ACE inhibitor	2303 (53.2)	549 (55.6)	176 (46.2)	128 (42.0)	<0.001
AT II inhibitor	570 (13.2)	153 (15.5)	89 (23.4)	75 (24.6)	<0.001
Calcium antagonist	409 (9.4)	92 (9.3)	57 (15.0)	52 (17.0)	<0.001
Statin	3760 (86.8)	815 (82.6)	292 (76.6)	223 (73.1)	<0.001
Diuretics	602 (13.9)	212 (55.6)	132 (34.6)	103 (33.8)	<0.001

Reference category: No anemia and no renal impairment.

### Transitions

Table 3 reports numbers of patients according to subgroup and stage, who experienced different transitions up to four years of follow-up. The most frequent transitions were PCI to ischemic events and PCI to death. The most probable transitions once having reached a specific stage were observed in patients with both anemia and renal impairment, with transition T5 from bleeding to death and T6 from ischemic events to death. If patients with both conditions experienced a bleeding event they had a 43% probability of death at four years (95% CI 25–63); if they experienced an ischemic event they had a 20% probability of death (95% CI 4–48). A similar, albeit attenuated pattern was observed for patients with renal impairment only, with a 39% probability of death in patients with a bleeding event (transition T5, 95% CI 20–61) and a 25% probability of death in patients with an ischemic event (transition T6, 95% CI 8–49).

**Table 3:** Transition-specific event rates by anemia and renal impairment, N = 6029.

Transitions	Reference	Anemiaonly	Renalimpairmentonly	Both anemiaand renalimpairment	p-value fordifferencesacross groups
**PCI (S1):**	**4346**	**990**	**384**	**309**	**<0.001**
T1: PCI to bleeding (S2)	99 (2.3)	56 (5.7)	23 (6.0)	30 (9.7)	
T2: PCI to ischemicevents (S3)	197 (4.5)	59 (6.0)	20 (5.2)	14 (4.5)	
T3: PCI to death (S4)	196 (4.5)	90 (9.1)	49 (12.8)	68 (22.0)	
No event	3854 (88.7)	785 (79.3)	292 (76.0)	197 (63.8)	
**Bleeding (S2):**	**99**	**56**	**23**	**30**	**0.005**
T4: Bleeding to ischemicevents (S3)	8 (8.1)	4 (7.1)	0 (0.0)	1 (4.3)	
T5: Bleeding to death (S4)	10 (10.1)	10 (17.9)	9 (39.1)	13 (43.3)	
No event	81 (81.8)	42 (75.0)	14 (60.9)	16 (53.3)	
**Ischemic events (S3):**	**205**	**63**	**20**	**15**	**0.019**
T6: Ischemic events todeath (S4)	18 (8.8)	1 (1.6)	5 (25.0)	3 (20.0)	
No event	187 (91.2)	62 (98.4)	15 (75.0)	12 (80.0)	

Reference category: No anemia and no renal impairment.

Displayed numbers in column 2–5 represent frequencies (%).

**Figure 2:**
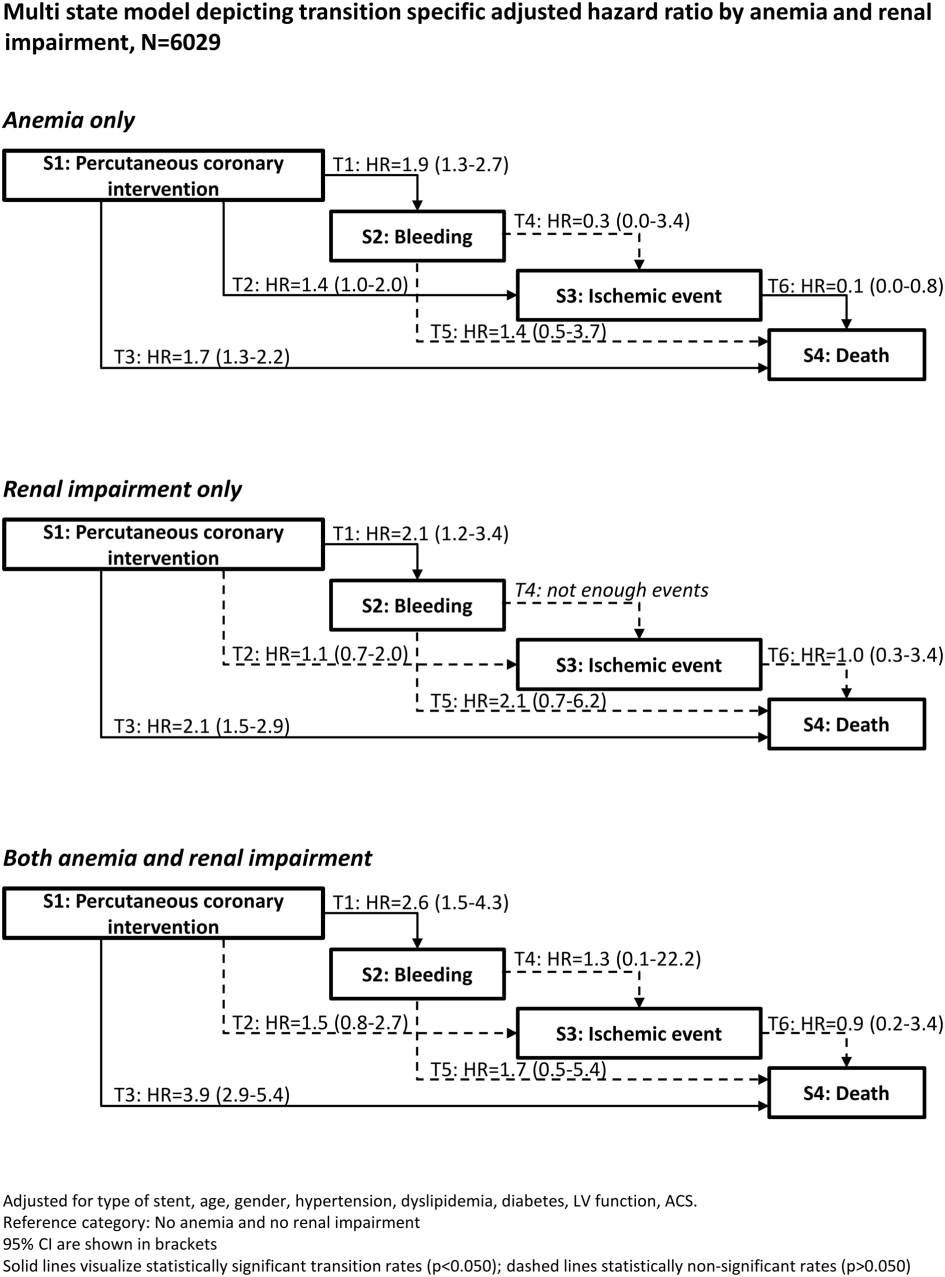
shows a multi-state model depicting transition-specific adjusted hazard ratios with 95% CI by anemia and renal impairment.

**Figure 3:**
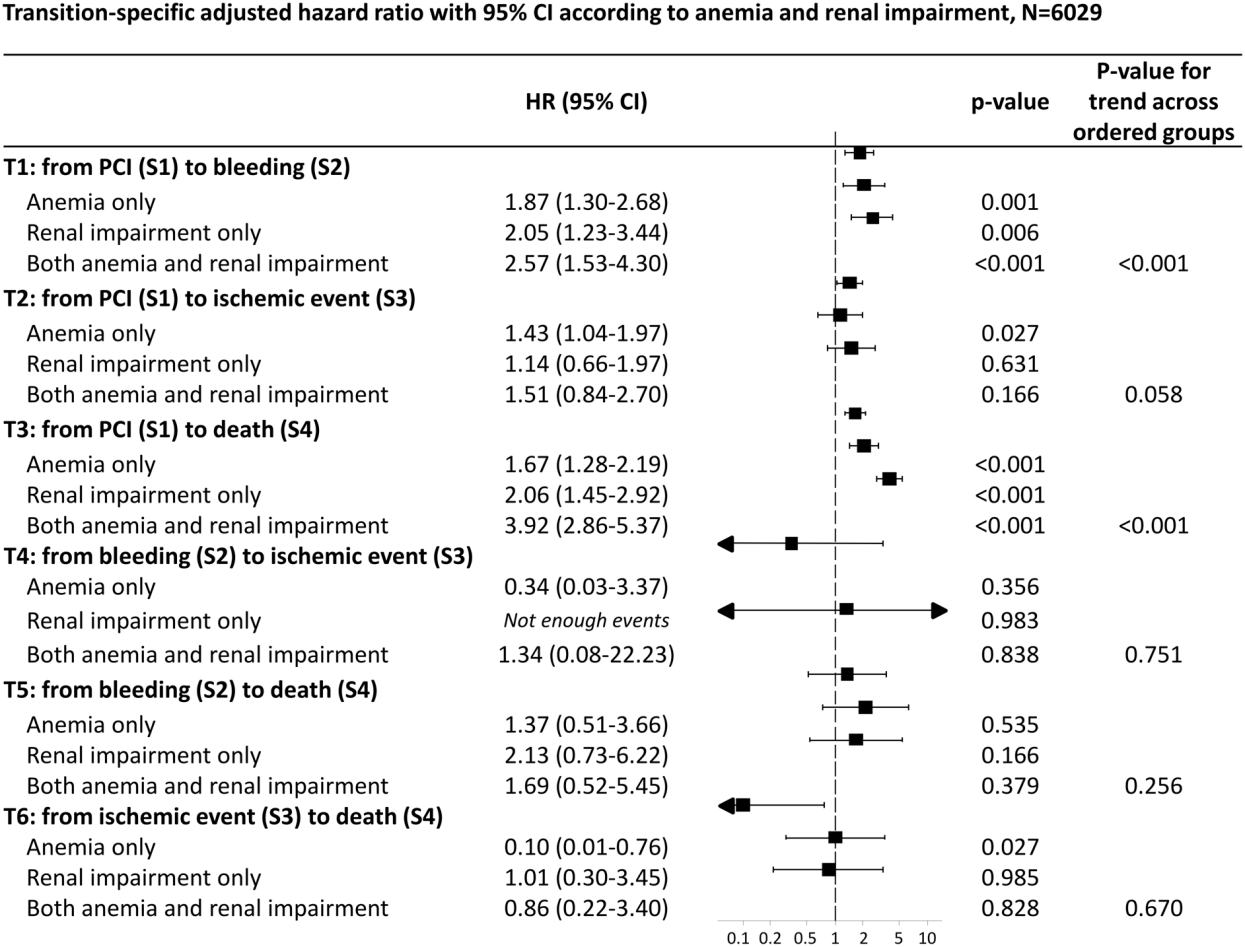
shows the transition-specific adjusted hazard ratios with 95% CI according to anemia and renal impairment using Cox proportional hazard models within a multi-state analysis. Adjustment for type of stent, age, gender, diabetes, hypertension, dyslipidemia, LV function, acute coronary syndrome. Reference: patients with no renal impairment and no anemia. P-value for trend estimated considering ordered groups.

Fig. 2 presents the multi-state model illustrating transition-specific adjusted hazard ratios for the three specific groups of patients with anemia only, renal impairment only, and anemia and renal impairment with the reference group. In patients with both conditions the risk of death (HR 3.9, 95% CI 2.9–5.4) was increased 4-fold as compared to the reference group and higher as compared to patients with either anemia or renal impairment alone. Hazard ratios indicated an increased risk for bleeding and death in all three groups compared to the reference group. Moreover, patients with anemia alone had a higher probability to have an ischemic event after PCI (HR 1.4, 95% CI 1.0–2.0) but a lower probability to die after an ischemic event compared to the reference group (HR 0.1, 95% CI 0.0–0.8). Fig. 3 summarizes the adjusted transition-specific hazard ratios for the three patient subgroups compared to the reference group.

### Additive effect of anemia and renal impairment

As described above, the additive effect of anemia and renal impairment is evaluated using different methods. In Fig. 3, we show that the additivity of anemia and renal impairment is statistically significant for the transition from PCI to bleeding (p<0.001), and from PCI to the primary outcome death (p<0.001), estimated using ordered groups. This trend across ordered groups is also illustrated in the corresponding forest plot. Table 4 presents further analyses of the additive effect of the two conditions. Both the AIC and the likelihood ratio tests of the comparison of the different models indicate that the additive model (model 3) has the best goodness of fit. This in turn is evidence that the effects of anemia and renal impairment are additive. Finally, using weighted random effects regression we show in Table 5 that the additivity of the two conditions can best be demonstrated for our primary outcome, death, as well as the composite of the events.

**Table 4:** Model comparison for the evaluation of additivity of anemia and renal impairment.

Model	AIC	χ^2^-statistic of model	p>χ^2^ of model	χ^2^-statistic of comparison	p>χ^2^ of comparison
1) Anemia only	6777.29	LR χ^2^ (1): 75.81	<0.001	LR χ^2^ (1) model 1 vs. null model: 75.81	<0.001
2) Renal impairment only	6750.89	LR χ^2^ (1): 102.21	<0.001	LR χ^2^ (1) model 2 vs. null model: 102.21	<0.001
3) Additive effect of anemia and renal impairment	6707.43	LR χ^2^ (2): 147.67	<0.001	LR χ^2^ (1) model 3 vs. model 2: 45.46	<0.001
4) Multiplicative interaction: anemia x renal impairment	6709.37	LR χ^2^ (3): 147.73	<0.001	LR χ^2^ (1) model 4 vs. model 3: 0.06	0.810

The first two models are compared to the null model, while model 3 is compared to model 2 (higher χ^2^-statistic as model 1) and model 4 to model 3 (higher χ^2^-statistic as model 2).

**Table 5:** Predicted probabilities and 95% CI.

Transitions	Reference	Anemia only	Renal impairment only	Both anemia and renal impairment	p-value for trend across ordered groups
To bleeding (S2)	1.9 (1.5–2.3)	3.9 (2.5–5.4)	3.8 (1.7–5.8)	5.4 (2.7–8.2)	0.068
To ischemic events (S3)	5.0 (4.3–5.8)	6.8 (5.1–8.4)	4.1 (1.9–6.3)	4.1 (1.4–6.8)	0.589
To death (S4)	6.2 (5.4–7.0)	12.7 (10.2–15.2)	20.2 (15.5–24.9)	32.2 (26.2–38.3)	0.009
Total events	13.1 (12.0–14.3)	23.4 (20.4–26.4)	28.0 (22.9–33.2)	41.8 (35.4–48.2)	0.009

Reference category: No anemia and no renal impairment.

Displayed numbers in column 2–5 represent predicted probabilities from multi-state Cox regression model (95% CI).

P-value for trend across ordered groups from weighted random effects regression.

### Estimated probabilities of clinical outcomes

**Figure 4:**
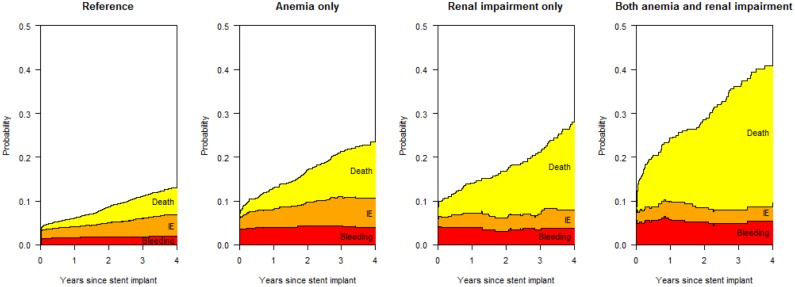
illustrates proportions of events up to four years according to anemia and renal impairment at baseline.

Fig. 4 illustrates the results of the multi-state model from index PCI up to four years of follow-up; the corresponding confidence intervals are displayed in Table 5. At four years, the probability of at least one event was 17.3% in the reference group without anemia nor renal impairment (95% CI 16.2–18.4), 23.4% in patients with anemia only (95% CI 20.4–26.4), 28.0% in patients with renal impairment only (95% CI 22.9–33.2) and 41.8% in patients with both anemia and renal impairment (95% CI 35.4–48.2).

## Discussion

We estimated the risk for specific transitions through intermediate adverse events that characterize progression of disease after PCI and modeled clinical outcomes as a function of anemia and renal impairment at baseline. The two conditions were encountered isolated or in combination in one in every four patients in a consecutive cohort of patients with coronary artery disease undergoing PCI. We found a strong gradient of risk for the composite of bleeding, ischemic events, or death as a function of hemoglobin value and estimated glomerular filtration rate at baseline. Presence of both, anemia and renal impairment increased the risk of clinical adverse events throughout four years after PCI to 42%. The risk of death was approximately 5-fold increased as compared to patients with neither anemia nor renal impairment, and almost doubled or tripled as compared to patients with either isolated renal impairment or anemia, respectively. Assessing the interaction between anemia and renal impairment we were able to show that the combination of the two conditions does not lead to an effect modification of one of the isolated conditions, but that their effects are additive.

The multi-state methodology applied in our analysis provides a holistic approach to clinical outcome assessment and accounts for progression of disease along a pathway through intermediate clinical events to eventual death. We observed a gradient of risk for clinical adverse events defined by the presence of the two conditions anemia and renal impairment isolated or in combination, respectively. Risk estimates were derived from an integrated analysis of all types of clinical outcome events and all occurring transitions from stage to stage. This approach is novel as compared to a traditional analysis, in which each outcome event is decontextualized, isolated from associated intermediate events. The magnitude of risk may thus be underestimated in traditional analysis. We avoided this limitation by modeling the progression of the disease through all stages.

Our analysis is weakened by a relatively small number of events per transition, in particular for secondary transitions from an intermediate event to eventual death, which made precise estimates of relative and absolute risks difficult for some of the transitions. Along the line, we found a lower risk of death after an ischemic event among patients with anemia only as compared to the reference group, which is likely to be related to the low number of observations for this secondary transition. In turn, the analysis was based on a consecutive cohort of unselected patients with nearly complete follow-up and centralized adjudication of clinical events.

Our analysis corroborates a high prevalence rate of anemia and renal impairment among patients with coronary artery disease. Approximately one quarter of patients undergoing PCI [1]–[3], [7] have been detected with renal impairment and/or anemia in previous reports. Both clinical entities are causally related to the development of coronary artery disease and myocardial ischemia, respectively, thereby explaining the high prevalence in this patient population. In addition, anemia and renal impairment were separately associated with increased long-term mortality following revascularization. Moderate renal impairment has been shown to increase all-cause mortality two-fold in a study of 11952 patients with coronary artery disease during a mean follow-up period of 3.6±2.2 years [7]. Severe anemia was associated with a similar mortality risk increase (HR 2.02, 95% CI 1.53–2.67) in a study of 6312 patients during a mean follow-up of 3.1±0.8 years [19]. In the present MSA we found a strong combined effect of anemia and renal impairment along a gradient of risk for clinical adverse events that was particularly driven by mortality.

## Conclusion

Applying a multi-state model we found evidence for a gradient of risk for the composite of bleeding, ischemic events, or death as a function of hemoglobin value and estimated glomerular filtration rate at baseline.
